# Common and distinct structural features of schizophrenia and bipolar disorder: The European Network on Psychosis, Affective disorders and Cognitive Trajectory (ENPACT) study

**DOI:** 10.1371/journal.pone.0188000

**Published:** 2017-11-14

**Authors:** Eleonora Maggioni, Benedicto Crespo-Facorro, Igor Nenadic, Francesco Benedetti, Christian Gaser, Heinrich Sauer, Roberto Roiz-Santiañez, Sara Poletti, Veronica Marinelli, Marcella Bellani, Cinzia Perlini, Mirella Ruggeri, A. Carlo Altamura, Vaibhav A. Diwadkar, Paolo Brambilla, ENPACT group

**Affiliations:** 1 Department of Neurosciences and Mental Health, IRCCS Fondazione Ca’ Granda Ospedale Maggiore Policlinico, University of Milan, Milan, Italy; 2 Department of Psychiatry, University Hospital Marqués de Valdecilla, School of Medicine, University of Cantabria-IDIVAL, Santander, Spain; 3 CIBERSAM, Centro Investigación Biomédica en Red Salud Mental, Santander, Spain; 4 Department of Psychiatry and Psychotherapy, Jena University Hospital, Jena, Germany; 5 Department of Psychiatry and Psychotherapy, Philipps University Marburg / Marburg University Hospital UKGM, Marburg, Germany; 6 Department of Clinical Neurosciences and Centro di Eccellenza Risonanza Magnetica ad Alto Campo, Scientific Institute and University Vita-Salute San Raffaele, Milan, Italy; 7 Department of Neurology, Jena University Hospital, Jena, Germany; 8 Department of Experimental and Clinical Medical Sciences (DISM), University of Udine, Udine, Italy; 9 Section of Psychiatry, Azienda Ospedaliera Universitaria Integrata Verona, Verona, Italy; 10 Department of Neurosciences, Biomedicine and Movement Sciences, Section of Clinical Psychology, University of Verona, Verona, Italy; 11 Department of Neurosciences, Biomedicine and Movement Sciences, Section of Psychiatry, University of Verona, Verona, Italy; 12 Department of Psychiatry & Behavioral Neuroscience, Wayne State University, Detroit, MI, United States of America; 13 IRCCS Scientific Institute “E. Medea”, Bosisio Parini, Lecco, Italy; 14 Department of Pathophysiology and Transplantation, University of Milan, Milan, Italy; King's College London, UNITED KINGDOM

## Abstract

**Introduction:**

Although schizophrenia (SCZ) and bipolar disorder (BD) share elements of pathology, their neural underpinnings are still under investigation. Here, structural Magnetic Resonance Imaging (MRI) data collected from a large sample of BD and SCZ patients and healthy controls (HC) were analyzed in terms of gray matter volume (GMV) using both voxel based morphometry (VBM) and a region of interest (ROI) approach.

**Methods:**

The analysis was conducted on two datasets, Dataset1 (802 subjects: 243 SCZ, 176 BD, 383 HC) and Dataset2, a homogeneous subset of Dataset1 (301 subjects: 107 HC, 85 BD and 109 SCZ). General Linear Model analyses were performed 1) at the voxel-level in the whole brain (VBM study), 2) at the regional level in the anatomical regions emerged from the VBM study (ROI study). The GMV comparison across groups was integrated with the analysis of GMV correlates of different clinical dimensions.

**Results:**

The VBM results of Dataset1 showed 1) in BD compared to HC, GMV deficits in right cingulate, superior temporal and calcarine cortices, 2) in SCZ compared to HC, GMV deficits in widespread cortical and subcortical areas, 3) in SCZ compared to BD, GMV deficits in insula and thalamus (p<0.05, cluster family wise error corrected). The regions showing GMV deficits in the BD group were mostly included in the SCZ ones. The ROI analyses confirmed the VBM results at the regional level in most of the clusters from the SCZ *vs*. HC comparison (p<0.05, Bonferroni corrected). The VBM and ROI analyses of Dataset2 provided further evidence for the enhanced GMV deficits characterizing SCZ. Based on the clinical-neuroanatomical analyses, we cannot exclude possible confounding effects due to 1) age of onset and medication in BD patients, 2) symptoms severity in SCZ patients.

**Conclusion:**

Our study reported both shared and specific neuroanatomical characteristics between the two disorders, suggesting more severe and generalized GMV deficits in SCZ, with a specific role for insula and thalamus.

## 1. Introduction

The neurobiological relationship between schizophrenia (SCZ) and bipolar disorder (BD) has been the subject of many debates but is still largely unknown [1–3]. Currently, the dimensional approach for diagnosing mental disorders has revitalized the question of shared characterization of SCZ and BD in a dimensional continuum view, as opposed to a categorical dichotomous vision. This has been supported by the neurobiological research that has shown that SCZ and BD exhibit shared genetic [4, 5] and neurocognitive determinants [6], besides clinical features [7]. The investigation of the neuroanatomical characteristics of the two disorders can therefore help to further delineate their common and different pathophysiological bases, opening the door to the development of tailored instruments with higher diagnostic specificity.

Meta-analyses of Magnetic Resonance Imaging (MRI) studies showed that both SCZ and BD are characterized by a global brain volume reduction, as well as by ventricular enlargement compared to healthy controls (HC) [1, 8, 9]. Voxel-Based Morphometry (VBM) meta-analyses reported gray matter volume (GMV) deficits in insula, thalamus, cingulate cortex, medial frontal gyrus, middle and superior temporal gyri in SCZ [8, 10], and in anterior cingulate cortex, inferior frontal gyrus, insula, middle and superior temporal gyri and pole and claustrum in BD [8, 11]. These studies suggest overlapping areas of GMV reduction among the two disorders, but also provide evidence for the larger extent of the deficits in SCZ than in BD [2, 8]. Conversely, only a minority of MRI studies directly compared BD and SCZ [3, 12–16]. A recent review on voxel-based comparisons between SCZ and BD found consistent evidence of GM deficits in SCZ compared to BD [17]. Region-based studies on SCZ *vs*. BD found in BD increased volume in the right amygdala and decreased volume in the bilateral ventricles compared to SCZ [1].

However, the meta-analyses that provided evidence for anatomical brain differences in patients with SCZ and BD were limited by the heterogeneity of the primary studies in terms of 1) clinical sample, 2) applied MR methodology and 3) post-processing techniques.

To provide robust information on the neuroanatomical correlates of SCZ and BD, the present work performs voxel-based and region-based analyses on a large dataset of 802 MR images recorded from HC, SCZ and BD patients across four European Research Centers, 1) the Jena University Hospital (Jena, Germany) (JUH), 2) the University Hospital Marqués de Valdecilla (Santander, Spain) (UHMV), 3) the Scientific Institute and University Vita-Salute San Raffaele (Milan, Italy) (UVSSR), 4) the University Hospital of Verona, (Verona, Italy) (VUH). These Centers, together with the University of Milan, Milan (Italy) (UNIMI), promote the European Network on Psychosis, Affective disorders and Cognitive Trajectory (ENPACT), whose purpose is to share and integrate clinical, demographic and neuroimaging data on BD and SCZ patients, as well as HC, in order to identify large-scale neuroanatomical similarities and differences associated with the two disorders. To our knowledge, this is one of the first studies comparing such a large sample of SCZ, BD and HC subjects in terms of GMV through voxel-based and region-based approaches.

## 2. Materials and methods

### 2.1. Subjects

Eight hundred and thirty-one subjects, 259 SCZ patients, 187 BD patients and 385 HC, were recruited across the four Centers. Preliminarily to the MRI acquisition, they signed a written informed consent to the protocol, in accordance with the Declaration of Helsinki and approved by the local ethical committee guidelines. After a quality check procedure, 802 subjects were selected for the analysis.

#### 2.1.1. Recruitment

In each Center, the diagnoses of BD and SCZ were assessed using the Structured Clinical Interview for Axis-I DSM Disorders (SCID) [18] and confirmed by the clinical consensus of an expert psychiatrist. In most of the cases, SCZ patients were scored using the Brief Psychiatric Rating Scale (BPRS), which was combined with the Positive and Negative Syndrome Scale (PANSS) (in Milan and Verona) or the Scales for Assessment of Positive/Negative symptoms (SANS/SAPS) (in Jena and Santander). In the majority of BD patients, depressive and manic symptoms were investigated using the Hamilton Rating Scale for Depression (HRSD) [19] and the Young Mania Rating Scale (YMRS) [20]. HC were recruited within the local communities of Jena, Milan, Santander and Verona through flyers and word of mouth. Subjects with personal or family history of psychiatric illnesses, personal history of substance or alcohol abuse, mental retardation or neurological disorders were excluded from the study.

### 2.2. MRI data acquisition

Structural T1-weighted images were recorded in the four Centers using 3.0 Tesla MRI scanners with the following parameters. JUH: Magnetization Prepared Rapid Gradient Echo (MPRAGE) sequence (matrix 256x256x192, 1 mm^3^ voxel), Siemens Tim Trio scanner (Siemens, Erlangen, Germany). UVSSR: MPRAGE sequence (matrix 256x256x220, 0.9x0.9x0.8 mm^3^ voxel), Philips Gyroscan Intera MR scanner (Philips, Best, the Netherlands). UHMV: T1-Fast Field Echo sequence (matrix 256x256x160, 0.94x0.94x1 mm^3^ voxel), Philips Intera scanner (Philips, Best, the Netherlands). VUH: MPRAGE sequence (matrix 256x256x160, 1 mm^3^ voxel), Magnetom Allegra Syngo scanner (Siemens, Erlangen, Germany).

### 2.3. MRI data processing

#### 2.3.1. Pre-processing

In a first quality check, the images affected by important inhomogeneity or movement artefacts were discarded. The images were then subjected to a non-parametric non-uniform intensity normalization (N3) using Freesurfer (http://surfer.nmr.mgh.harvard.edu) [21]. This preliminary step was specifically added to remove non-uniformity artifacts and homogenize the image intensities across sites. The bias-corrected images were exported to Matlab R2014A (The Mathworks, Inc®) for the following analyses. The image pre-processing was carried out using Statistical Parametric Mapping (SPM), version 12 (http://www.fil.ion.ucl.ac.uk/spm/software/spm12/), and its VBM8 toolbox (http://www.neuro.uni-jena.de/vbm/download/). The images were segmented using SPM12 segmentation, which performs bias regularization and classifies the images into gray matter (GM), white matter (WM), cerebrospinal fluid, bone, soft tissue and air/background. In this procedure, the GM and WM images of the subjects were also rigidly aligned via an affine transformation. The Dartel (Diffeomorphic Anatomical Registration Through Exponentiated Lie algebra) tools were then used to determine the nonlinear deformations for registering the GM and WM images of all subjects. Finally, the registered images were normalized to MNI space and smoothed with a 6 mm Full Width Half Maximum (FWHM) Gaussian kernel. A final quality check on the pre-processed images based on 1) visual inspection and 2) covariance homogeneity was performed using VBM8 modules: the images with an overall covariance below two standard deviations were discarded from the analysis. The VBM and ROI analyses (2.3.2 and 2.3.3 sections) were applied to both datasets. The results on Dataset2 are described in a Supplementary section, S1 Results.

#### 2.3.2. VBM comparison across diagnostic groups

The VBM analysis was performed on the pre-processed GM images using SPM12. The VBM analysis of each dataset was performed using a full-factorial General Linear Model (GLM) design with two factors, the diagnosis factor with 3 levels (SCZ, BD and HC) and the scanning site factor with four levels (Jena, Milan, Santander, Verona). While the diagnosis represented the factor of interest, the center factor was included with the purpose to discard its contribution. The measurements were assumed to be independent and with unequal variance between levels. Age and gender were included as covariates, both interacting with the factor diagnosis. The volumetric differences among subjects were considered by proportional scaling for the total intracranial volume (ICV). A GM mask with optimal threshold for GMV was created using the masking toolbox (http://www0.cs.ucl.ac.uk/staff/g.ridgway/masking/) and used in the GLM analysis. Inference on the GMV differences between groups was made using double-sided t-tests (p<0.05, cFWE corrected). A conjunction analysis was performed to extract the GMV reductions common to SCZ and BD. A direct SCZ-BD comparison was also conducted.

#### 2.3.3. ROI comparison across diagnostic groups

The ROI analysis was performed on the anatomical regions containing the significant VBM clusters (p<0.05, cFWE corrected), with the main objective to verify whether the local GMV differences emerged from the VBM comparison were still significant at the global level in the corresponding anatomical regions. The anatomical location of the VBM clusters resulting from the pairwise t-contrasts (paragraph 2.3.2) was identified using the Automated Anatomical Labeling (AAL) atlas [22]. The volume of the selected AAL regions was estimated by summing up the GMV of the voxels within the entire anatomical region. In the GLM analyses, performed using in-house Matlab scripts, the AAL regional volume was modeled in terms of diagnosis, scanning site, age, gender and ICV. The differences between groups were investigated using double-sided t-tests. To limit the false positive rates, a very conservative multiple comparison correction was applied by using the Bonferroni method with N = 116, corresponding to the number of regions within the AAL atlas. The significance threshold was set to p = 0.05, Bonferroni corrected.

#### 2.3.4. Clinical-neuroanatomical correlations

In the patient groups, we investigated the possible effects of clinical variables on GMV. All the clinical variables were analyzed in separate designs, including case by case the subsets of patients having the information of interest. More details on scores and availability of clinical information can be found in Table 1.

**Table 1:** Demographic and clinical details in the two datasets. In Dataset1, from the analysis of variance (ANOVA), significant age differences across groups emerged (p<0.01). Sex ratio (Males/Females) was 1.67 in SCZ, 0.64 in BD and 1.04 in HC (χ^2^ = 23.02, p<0.0001).

**Dataset1**	**SCZ PROBANDS (N = 243)**	**BD PROBANDS (N = 176)**	**HC (N = 383)**
**Demographic characteristics**			
**Age (years) (mean ± SD)**	33.24 ± 9.41	44.7 ± 12.08	30.4 ± 9.2
**Male gender (N, %)**	152, 62.6%	69, 39.2%	195, 50.9%
**Site of acquisition (N, %)**	Jena (45, 18.5%), Milan (82, 33.74%), Santander (89, 36.63%), Verona (27, 11.11%)	Jena (23, 13.07%), Milan (134, 76.14%), Santander (0), Verona (19, 10.8%)	Jena (111, 28.98%), Milan (74, 19.32%), Santander (105, 27.42%), Verona (93, 24.28%)
**Clinical characteristics**			
**Age of onset (N, mean ± SD)**	229, 26.16 ± 7.65	150, 30 ± 10	n.a.
**Psychosis, yes**	243 (100%)	42 (28.86%)	0 (0%)
**Antipsychotics, yes**	233 (95.88%)	57 (32.39%)	0 (0%)
**Mood stabilizers (N, N yes)**	144, 6 (2.47%)	139, 86 (48.86%)	383, 0 (0%)
**Benzodiazepines (N, N yes)**	155, 110 (45.27%)	131, 94 (53.41%)	383, 0 (0%)
**BD type**	n.a.	BD-I: 111 (63.07%), BD-II: 54 (30.68%), no information: 11 (6.25%).	n.a.
**Mood state (N, %)**	n.a.	Euthymic (23, 13.07%), hypomanic (1, 0.57%), mixed (1, 0.57%), depressed (120, 68.18%), manic (27, 15.34%), no information (4, 2.27%)	n.a.
**Psychopathology**			
**HRSD (N, mean ± SD)**	n.a.	107, 17.49.13 ± 9.61	n.a.
**BPRS (N, mean ± SD)**	220, 50.46 ± 16.12	n.a.	n.a.
**PANSS (N, mean ± SD)**	95, 73.38 ± 18.59	n.a.	n.a.
**SAPS (Center, N, mean ± SD)**	Jena: 44, 20.21 ± 11.48. Santander: 89, 14.14 ± 3.92.	n.a.	n.a.
**SANS (Center, N, mean ± SD)**	Jena, 44, 42.43 ± 14.15. Santander: 89, 5.84 ± 5.81.	n.a.	n.a.
**Dataset2**	**SCZ PROBANDS (N = 109)**	**BD PROBANDS (N = 85)**	**HC (N = 107)**
**Demographic characteristics**			
**Age (years) (mean ± SD)**	39.1 ± 8.78	39.13 ± 9.9	39.02 ± 10.26
**Male gender (N, %)**	67, 61.47%	34, 40%	52, 48.6%
**Site of acquisition (N, %)**	Jena (19, 17.43%), Milan (33, 30.28%), Santander (42, 38.53%), Verona (15, 13.76%)	Jena (22, 25.88%), Milan (53, 62.35%), Verona (10, 11.76%)	Jena (21, 19.63%), Milan (26, 24.3%), Santander (46, 42.99%), Verona (17, 15.89%)
**Clinical characteristics**			
**Age of onset (N, mean ± SD)**	102, 30 ± 8.12	63, 28.08 ± 8.94	n.a.
**Psychosis, yes**	109 (100%)	26 (30.59%)	0 (0%)
**Antipsychotics, yes**	105 (96.33%)	33 (38.82%)	0 (0%)
**Mood stabilizers (N, N yes)**	67, 5 (4.58%)	59, 41 (48.24%)	383, 0 (0%)
**Benzodiazepines (N, N yes)**	74, 49 (44.95%)	57, 38 (44.71%)	107, 0 (0%)
**BD type**	n.a.	BD-I: 54 (63.53%), BD-II: 28 (32.94%), no information: 3 (3.53%).	n.a.
**Mood state (N, %)**	n.a.	Euthymic (22, 25.89%), hypomanic (1, 0.57%), mixed (0, 0%), depressed (49, 57.65%), manic (12, 14.12%), no information (1, 1.18%)	n.a.
**Psychopathology**			
**HRSD (N, mean ± SD)**	n.a.	66, 14.52± 10.68	n.a.
**BPRS (N, mean ± SD)**	99, 50.02 ± 15.56	n.a.	n.a.
**PANSS (N, mean ± SD)**	42, 70.64 ± 17.58	n.a.	n.a.
**SANS (Center, N, mean ± SD/median)**	Jena: 19, 42.21 ± 16.42. Santander: 42, 4.5	n.a.	n.a.
**SAPS (Center, N, mean ± SD)**	Jena: 19, 19.84 ± 10.19. Santander: 42, 13.60 ± 3.37	n.a.	n.a.

SCZ: schizophrenia. BD: bipolar disorder. HC: healthy controls. HRSD: Hamilton Depression Rating Scale. BPRS: Brief Psychiatric Rating Scale. PANSS: Positive And Negative Syndrome Scale. SAPS/SANS: Scales for Assessment of Positive/Negative symptoms. SD: standard deviation. The median value is indicated in case of non-normal distribution.

In the BD sample, we analyzed the contribution of HRSD, BD type, psychotic features and therapy based on benzodiazepines, antipsychotics and mood stabilizers. In the SCZ sample, we investigated the contribution of BPRS, PANSS, SAPS, SANS, duration of untreated illness and therapy with benzodiazepines. Since SAPS/SANS scores were not uniformly distributed between Jena and Santander, their effects were studied in one center at a time. The possible impact of age of onset on GMV was separately investigated in the two groups of patients.

The contribution of each clinical parameter on GMV was evaluated first at the voxel level, using VBM, and then at the regional level, considering the significant AAL regions/clinical variables from the VBM study. The ROI study was performed to verify the significance of the clinical-neuroanatomical relation at the regional level.

In the VBM GLM analyses, the GMV of each voxel was modeled in terms of the clinical variable of interest, age and gender. Since some variables were available across different centers, in the corresponding models we included the site factor to remove its contribution. As in the main VBM analysis (section 2.3.2), we accounted for the effect of head volume by proportional scaling with ICV and we masked the results using the optimal threshold GM mask. Inference on the contribution of the variable of interest was performed using double-sided t-tests (p<0.001, >100 voxels).

In the ROI analyses, using in-house Matlab scripts, we conducted Pearson linear partial correlation analyses between regional GMV (normalized for ICV) and the clinical variable of interest, while accounting for the contribution of age and gender and, when appropriate, scanning site. The significance threshold was set to p = 0.01.

## 3. Results

### 3.1. Demographic and clinical information

The demographic and clinical details of the two datasets are reported in Table 1.

Dataset1 was composed of 383 HC (195 males, range 18–62 years, 30.4 ± 9.2 years), 243 patients with SCZ (152 males, range 17–61 years, 33.24 ± 9.41 years) and 176 patients with BD (69 males, range 20–76 years, 44.7 ± 12.08 years). Both psychotic and non-psychotic BD patients (PBD, NPBD) as well as BD type I (BD-I) and BD type II (BD-I) patients took part to the study: PBD patients were in minority, being 42 out of 176, whereas BD-I patients were in majority, being 111 out of 176. Since no significant GMV differences emerged between them (more details are in paragraph 3.3), in the main analysis BD patients were considered in a unique group. From the analysis of variance (ANOVA), significant age differences across BD, SCZ and HC emerged (p<0.01). Sex ratio (M/F) was 1.67 in SCZ, 0.64 in BD and 1.04 in HC (χ^2^ = 23.02, p<0.0001). The fact that SCZ is diagnosed 1.4 times more frequently in males than in females [23] supports the SCZ gender ratio. On the other hand, the women prevalence in the BD group may be explained by the fact that BD patients were mostly recruited as depressed inpatients, as women with BD may have more depressive and mixed episodes than do men with the illness.

At the time of the MRI study, 23 BD patients were euthymic, 1 was in hypomanic state, 1 had mixed episodes, 27 were in manic phase and 120 were in depressed phase. This information was missing in 4 patients. The assessment of euthymic, manic and depressed states was supported by HRSD and YMRS scores. Indeed, in manic patients the mean YMRS score was 20.8, in depressed patients the mean HDRS score was 22.32, whereas in euthymic ones both HDRS and YMRS were below 8. Overall, the prevalence of patients in depressed phase is confirmed by the mean HRSD score, which was above 17 (as reported in Table 1).

In the SCZ sample, the mean BPRS and positive and negative symptoms rates from PANSS or SANS/SAPS suggest that patients from all centers were from moderately to markedly ill [24, 25].

Concerning medication, antipsychotics were assumed by most SCZ patients (95.88%) and by less than half of BD patients (32.39%). Among SCZ patients, 3 were treated with amisulpride, 49 with aripiprazole, 42 with clozapine, 13 with haloperidol, 14 with olanzapine, 6 with paliperidone, 25 with quetiapine, 32 with risperidone, 17 with ziprasidone; this information was missing for 30 patients. Among BD patients taking neuroleptics, 2 were treated with haloperidol, 4 with amisulpride, 2 with aripiprazole, 1 with chlorpromazine, 1 with clozapine, 11 with haloperidol, 5 with olanzapine, 1 with paliperidone, 15 with quetiapine and 7 with risperidone; this information was missing for 7 patients. From the available information on benzodiazepines, we know that BD patients taking benzodiazepines were in majority (at least the 53%). This drug was used also in the therapy of around one half of SCZ patients (45% or more). Mood stabilizers were included in the therapy of approximately half of the BD sample (at least the 48%) but were almost never used in the treatment of SCZ patients (≈2%).

A reduced dataset (Dataset2), more homogeneous in terms of age and diagnosis, was manually extracted from the original one for a further analysis, with the main objective to investigate the neuroanatomical differences among SCZ, BD and HC in a subset of Dataset1 without significant age differences across groups. Dataset2 included 107 HC (52 males, 20–62 years, 39.02 ± 10.26 years), 85 BD patients (34 males, 20–60 years, 39.13 ± 9.9 years) and 109 SCZ patients (67 males, 20–61 years, 39.1 ± 8.78 years). Since each group of patients was reduced by more than half, from the analysis of Dataset2 we did not expect to reproduce the findings from the main dataset, but we wanted to extract the most relevant features of the disorders. Compared to Dataset1, the balance between psychotic *vs*. non-psychotic BD patients and BD-I *vs*. BD-II patients was preserved; again, the depressed patients outnumbered the euthymic and manic ones. Concerning the SCZ sample, the mean BPRS, SAPS/SANS and PANSS scores still indicated moderate to severe schizophrenic symptoms.

Concerning medication, antipsychotics were taken by 96.33% of the SCZ sample and 38.82% of the BD sample. Among SCZ patients, 1 used amisulpride, 26 aripiprazole, 15 clozapine, 7 haloperidol, 5 olanzapine, 4 paliperidone, 9 quetiapine, 15 risperidone and 9 ziprasidone; the antipsychotic type was not known in 14 patients. Among BD patients, 1 used aripiprazole, 1 chlorpromazine, 9 haloperidol, 4 olanzapine, 10 quetiapine and 4 risperidone, whereas the neuroleptic type was not known in 4 patients. Benzodiazepines were used by almost half of both BD and SCZ patients (≈45%). Mood stabilizers were included in the therapy of around half of BD patients (48% or more) but were almost unused in SCZ patients (less than 5%).

### 3.2. Comparison among diagnostic groups

The VBM and ROI results of Dataset1 are separately reported in sections 3.2.1 and 3.2.2, respectively. The VBM and ROI results of Dataset2 are described in S1 Results.

#### 3.2.1. VBM analysis

**Fig 1:**
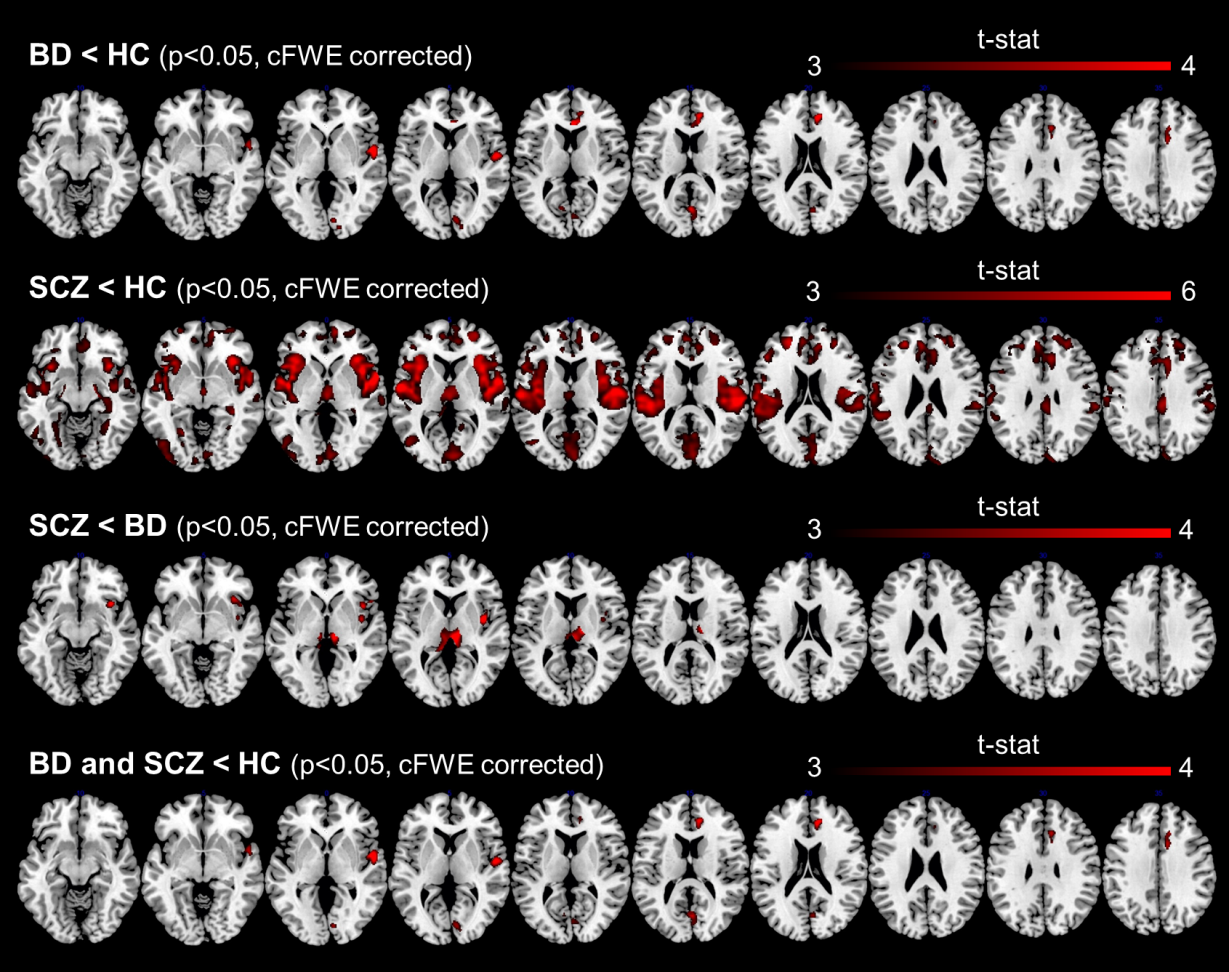
Results of the VBM analysis of Dataset1: regions with significant GMV difference between SCZ, BD and HC (p<0.05, cFWE corrected). VBM: voxel based morphometry. GMV: gray matter volume. SCZ: schizophrenia. BD: bipolar disorder. HC: healthy controls. cFWE: cluster family wise error.

The VBM results of Dataset1 are reported in Fig 1, where the clusters with significant GMV difference between the three couples of groups are shown. The anatomical location and statistics of the SPM peaks (the first of each cluster) and the corresponding cluster extension are listed in Table 2. Compared to HC, BD patients showed reduced GMV (p<0.05, cFWE corrected) in three areas, mainly in the right hemisphere. These regions were located in 1) anterior and mesial portions of the cingulate cortex, 2) superior temporal cortex and temporal pole, 3) calcarine cortex, cuneus and lingual gyrus. The peak voxel was in the right subgenual cingulate cortex.

**Table 2:** Statistics of the significant ROIs (p<0.05, cFWE corrected) of the VBM analysis of Dataset1. Degrees of freedom: [1 785]. t-stat threshold: 3.1.

T CONTRAST	CLUSTER P(FWE)	# VOXELS	PEAK T	X,Y,Z [22]	AAL REGION
**BD < HC**	0.001	1152	4.79	3, 32, 8	Anterior cingulate cortex, R
	0.042	544	4.53	54, 0, 0	Superior temporal cortex, R
	0.026	622	3.92	2, -69, 15	Calcarine cortex, R
**SCZ < HC**	<0.001	20010	7.50	-57, -6, 5	Superior temporal cortex, L
	<0.001	19855	6.74	56, 2, 2	Superior temporal cortex, R
	<0.001	12948	5.84	5, -30, 44	Mid cingulate cortex, R
	<0.001	2255	4.93	-51, -57, -15	Inferior temporal cortex, L
	<0.001	4635	4.91	5, -86, 3	Calcarine cortex, R
	0.002	1071	4.12	-38, -69, -51	Cerebelum 7b, L
	0.014	718	3.55	-23, -78, -47	Cerebelum Crus 2, R
**BD+SCZ < HC**	0.042	544	4.53	54, 0, 0	Superior temporal cortex, R
	0.001	833	4.41	3, 32, 8	Anterior cingulate cortex, R
	0.026	520	3.83	8, -83, 2	Calcarine cortex, R
**SCZ < BD**	0.009	789	4.47	39, -2, 5	Insula, R
	0.001	1246	4.46	5, -26, 6	Thalamus, R

BD: bipolar disorder. SCZ: schizophrenia. HC: healthy controls. cFWE: cluster family wise error. AAL: Automated Anatomical Labeling. R: right hemisphere, L: left hemisphere.

Compared to HC, SCZ patients showed widespread GMV reductions throughout the cortex (p<0.05, cFWE corrected). The GMV deficits were detected in the frontal (anterior and mid cingulate cortex, rectus, frontal superior medial and mid frontal cortex), parietal (inferior parietal cortex, supramarginal and postcentral gyri), occipital (cuneus and calcarine cortex) and temporal cortices (mid and superior temporal cortex and pole, rolandic operculum, Heschl’s gyrus, insula) of both hemispheres. An extensive GMV reduction also spanned bilaterally the thalamus, hippocampus, parahippocampal gyrus and amygdala, as well as portions of the cerebellum. The most significant voxel was in the left superior temporal cortex.

The conjunction analysis revealed shared GMV deficits in SCZ and BD patients compared to HC (p<0.05, cFWE corrected) in three clusters located in the superior temporal cortex, anterior and mid cingulate cortex and occipital cortex of the right hemisphere. Although the areas of GMV deficit of SCZ patients mostly included the BD ones, the subgenual portion of the right cingulate cortex emerged only in BD patients.

The comparison between BD and SCZ revealed significant GMV differences in the bilateral thalami and in the right insula, which showed GMV deficits in SCZ patients compared to BD patients (p<0.05, cFWE corrected).

#### 3.2.2. ROI analysis

**Fig 2:**
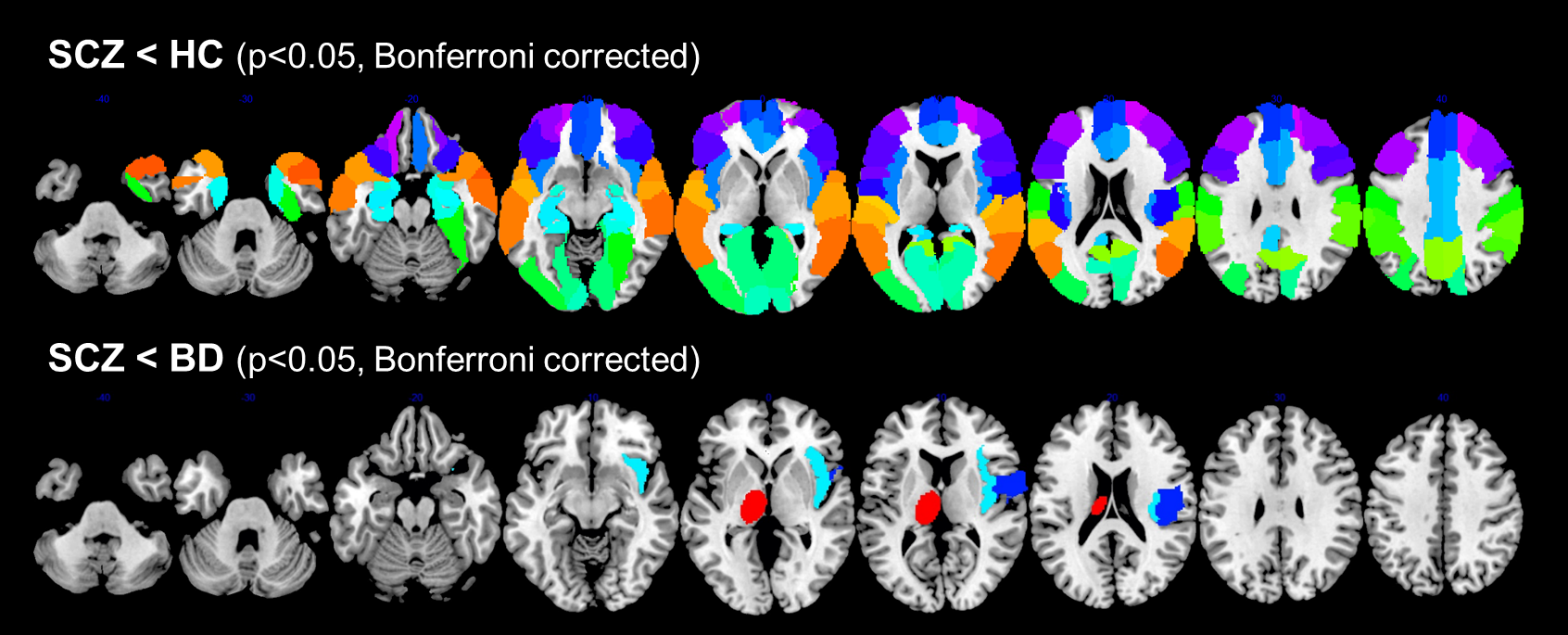
Results of the ROI analysis of Dataset1. **AAL regions with significant GMV differences between SCZ, BD and HC (p<0.05, Bonferroni corrected).** ROI: region of interest. AAL: Automated Anatomical Labeling. GMV: gray matter volume. SCZ: schizophrenia. BD: bipolar disorder. HC: healthy controls.

The AAL regions with global GMV difference between the different groups (SCZ *vs*. HC, BD *vs*. HC, SCZ *vs*. BD, SCZ and BD *vs*. HC) are reported in Table 3 and shown in Fig 2 (p<0.05, Bonferroni corrected). Overall, at such conservative significance threshold, the local GMV abnormalities were confirmed at the regional level in SCZ patients but not in BD ones.

**Table 3:** Significant AAL regions of the ROI analysis of Dataset1 (p<0.05, Bonferroni corrected).

T CONTRAST	AAL CLUSTERS LEFT HEMISPHERE	AAL CLUSTERS RIGHT HEMISPHERE
**SCZ < HC**	Frontal cortex, inferior and mid orbitofrontal cortex, rolandic operculum, insula, cingulate cortex, hippocampus, parahippocampal gyrus, amygdala, calcarine cortex, precuneus, lingual gyrus, inferior and mid occipital cortex, postcentral gyrus, inferior parietal cortex, supramarginal gyrus, Heschl’s gyrus, mid and superior temporal cortex, superior temporal pole.	Inferior and mid frontal cortex, orbitofrontal cortex, rolandic operculum, rectus, insula, anterior and mid cingulate cortex, hippocampus, parahippocampal gyrus, amygdala, calcarine cortex, precuneus, cuneus, lingual gyrus, fusiform gyrus, postcentral gyrus, inferior parietal cortex, supramarginal gyrus, Heschl’s gyrus, mid and superior temporal cortex, temporal pole.
**BD < HC**	/	/
**SCZ < BD**	Thalamus	Rolandic operculum, insula.
**SCZ AND BD < HC**	/	/

BD: bipolar disorder. SCZ: schizophrenia. HC: healthy controls. ROI: region of interest. AAL: Automated Anatomical Labeling.

Indeed, the local GMV deficits in SCZ compared to HC (VBM t-contrast: SCZ<HC, p<0.05, cFWE corrected) were confirmed at the regional level with a high level of significance (p<0.05, Bonferroni corrected) in the majority of AAL regions, 57 out of 88, with the most significant deficits in the bilateral rolandic operculum, insula, Heschl’s gyrus and superior temporal cortex, and in the left supramarginal gyrus and triangular inferior frontal cortex.

With regard to the GMV deficits in BD compared to HC (VBM t-contrast: BD<HC, p<0.05, cFWE corrected), the ROI analysis did not confirm the VBM results at the regional level in any of the regions. Accordingly, the shared GMV deficits of BD and SCZ compared to HC were not confirmed at the regional level.

Among the clusters showing lower GMV in SCZ compared to BD (VBM t-contrast: SCZ<BD, p<0.05, cFWE corrected), the ROI analysis confirmed the VBM results in the right rolandic operculum, right insula and left thalamus (p<0.05, Bonferroni corrected).

### 3.3. Clinical-neuroanatomical correlations

The results of the neuroanatomical-clinical correlation analyses are reported in Table 4 (p<0.001, >100 voxels).

**Table 4:** Statistics of the VBM analysis of clinical variables. Degrees of freedom: [1 785]. t-stat threshold: 3.1.

VARIABLE	DIAGNOSIS	# VOXELS	T STAT	P-VALUE	X,Y,Z [22]	AAL REGION
**MOOD STABILIZERS**	BD	180	4.05	<0.001	-8, -9, 11	Left thalamus
		111	3.80	<0.001	-11, -42, -6	Left cerebellum
**BENZODIAZEPINES**	BD	151	-4.21	<0.001	-29, 20, 44	Left midfrontal cortex
		134	-3.93	<0.001	39, 11, 26	Right inferior frontal cortex, opercular portion
		610	-3.90	<0.001	9, -95, -2	Right calcarine cortex
		243	-3.83	<0.001	-3, -12, 62	Left supplementary motor cortex
**AGE OF ONSET**	BD	242	4.40	<0.001	-60–20–8	Left midtemporal cortex
**AGE OF ONSET**	BD	257	4.10	<0.001	21 12 53	Right superior frontal cortex
**SANS (JENA)**	SCZ	106	-3.89	<0.001	-32, 15, -23	Left superior temporal pole
**SAPS (SANTANDER)**	SCZ	218	-4.34	<0.001	-27, -54, 38	Left inferior parietal cortex

BD: bipolar disorder. SCZ: schizophrenia. AAL: Automated Anatomical Labeling. SANS: Scale for Assessment of Negative Symptoms. SAPS: Scale for Assessment of Positive Symptoms.

In BD patients, the contributions of HRSD scores, BD type, psychotic features and antipsychotic therapy on GMV were not significant. However, we found a positive association between age of onset and GMV in two clusters belonging to right superior frontal cortex and left mid temporal cortex. We also found neurotrophic effects of mood stabilizers, which were associated with higher GMV in thalamus and cerebellum in the left hemisphere. On the contrary, BD patients treated with benzodiazepines showed reduced GMV in bilateral frontal cortex (medial portion in the left hemisphere, inferior opercular portion in the right one), right calcarine cortex and left supplementary motor cortex. The ROI analysis confirmed this association at the regional level in right inferior frontal cortex (opercular portion) and right calcarine cortex (p<0.01).

In SCZ patients, no significant effects of BPRS scores, therapy with benzodiazepines, duration of untreated illness and age of onset emerged. In the subset of patients scored with PANSS, we did not find any GMV correlates of the scale scores. However, in SCZ patients from Jena, we found a negative association between SANS scores and GMV in left superior temporal pole. In patients from Santander, a negative relation emerged between SAPS scores and GMV in left inferior parietal cortex. These results were not confirmed at the regional level.

## 4. Discussion

In the present work, we integrated and quantitatively analyzed a very large sample of structural MRI data of HC and patients with SCZ and BD. Our ultimate objective was to obtain a robust large-scale identification of the structural cerebral differences associated with the two disorders, overcoming the limitations related to low sample size and statistical power that affect most of the current studies on SCZ and BD.

Our results show that the partial overlap between BD and SCZ in terms of clinical phenotype may rely on shared neuroanatomical changes, supporting the hypothesis of common brain structure endophenotypes across categorical diagnoses [26]. However, our findings also show points of difference across the two diagnoses and demonstrate the greater extent of the SCZ pathology, associated with brain structures that are more severely compromised in comparison with BD. The increased volumetric deficits in SCZ patients may be related to the more severe clinical picture and cognitive impairment currently seen in SCZ than in BD [27–29]. Although clinical manifestations and cognitive deficits can be significant in BD too, in our BD sample the prevalence of non-psychotic patients may have contributed to widen the GMV differences between BD and SCZ.

### 4.1. Gray matter phenotypes of SCZ and BD

The analyses conducted in this multicentric study revealed overlapping volume deficits in the two groups of patients, being more severe and generalized in SCZ. The VBM results on Dataset1 from the SCZ-BD conjunction analysis showed in the two patient groups compared to HC a GM atrophy in superior temporal cortex and temporal pole, anterior and mid cingulate cortex and calcarine cortex of the right hemisphere; the shared GMV reduction in right temporal cortex, more pronounced in SCZ, was confirmed by the analyses on Dataset2. In the latter analysis, the emergence of GMV deficits in BD only at the uncorrected significance level may be the effect of the removal of many older patients from the BD sample. Overall, our findings are consistent with the ones of previous studies, showing in SCZ and BD overlapping deficits in cingulate cortex and temporal lobe [8, 14].

Previous studies using different MRI contrasts have confirmed the key role of these regions in these disorders. Reduced WM volume, fractional anisotropy and GM complexity in frontal and temporo-parietal regions emerged in both SCZ and BD [15, 30–35]. The cortical thickness, cortical volume, water diffusion and blood perfusion in fronto-temporo-limbic regions have also emerged as relevant features for classification of first episode psychosis (FEP) [36–38]. Functional MRI studies reported dysfunctional superior temporal gyrus and disrupted fronto-temporal connectivity in patients with FEP and in ultra-high-risk subjects [39, 40]. Furthermore, the dorsal anterior cingulate cortex represents a key node of the cingulo-opercular network, exerting top-down control over sensory and limbic regions, which emerged to be disconnected in patients with SCZ [41] and BD [42]. More in general, global structural and functional connectivity metrics have been shown to be abnormal in psychotic illnesses [43].

### 4.2. Specific characteristics of SCZ

In line with previous studies [8, 12, 44], our VBM and ROI findings confirm the larger extent and magnitude of GMV deficits in SCZ patients than in BD patients when compared to HC, with the regions of GMV reduction in BD largely included in the SCZ ones.

The VBM analyses of both datasets revealed widespread deficits of SCZ patients, which appeared to be highly significant and symmetrically distributed across the two hemispheres. The ROI analyses strengthened the above results, providing evidence for the consistence of such deficits at the regional level in SCZ but not in BD. While the local GMV deficits associated with BD were mainly in the right hemisphere, the SCZ ones spread to the left hemisphere. The right frontal and temporal areas showing lower GMV in SCZ compared to HC were more extended than BD ones, including the rolandic operculum, Heschl’s gyrus, insula, dorsolateral prefrontal cortex and wider portions of the cingulate cortex. Noticeably, in SCZ the left temporal deficits were more significant than the right ones: this finding, besides being supported by wide literature evidence [10, 45, 46], may be related to the key role of left temporal cortex in auditory information processing, hallucinations and thought disorders.

The VBM analyses also highlighted thalamic, hippocampal and amygdalar deficits in SCZ patients but not in BD ones. The anatomical abnormalities of these structures in SCZ patients appear to be consistent with their extensive cognitive deficits [28, 47, 48]. In line with the hypothesis of “cognitive dysmetria”, the GMV reduction in key nodes of information processing may underlay the deficits in integrating and coordinating information of SCZ [49]. The VBM and ROI results on Dataset2 confirmed the extended volumetric deficits characterizing SCZ compared to HC, involving both cortical and subcortical regions.

The areas of GMV reduction in SCZ highlighted in our study were consistently reported in previous voxel-based [8, 10, 15, 44, 50, 51] and region-based studies, showing GM changes in, for example, anterior cingulate cortex [52], superior temporal gyrus [53], insula [54], entorhinal cortex [55] and amygdala [47]. Changes in WM have also been reported in SCZ patients [27], mainly in frontal and temporo-occipital areas [46, 56], corpus callosum [46, 57, 58] and thalamus [59].

### 4.3. Specific characteristics of BD

Despite the wider deficits characterizing SCZ in comparison with HC, the VBM analysis of Dataset1 highlighted a specific deficit of BD patients compared to HC in a portion of the right anterior cingulate cortex, crossing the perigenual and subgenual areas. This specific deficit of BD was already described [8] and is in line with postmortem studies on BD, showing in this region decreased neural density and thickness of cortical layers III, V and VI [60]. Since the subgenual anterior cingulate cortex is implicated in the processing and modulation of emotions, its specific abnormality in BD patients may be interpreted on the light of the emotional dysregulation that characterizes mood disorders [61, 62]. Regional GMV deficits in these areas have been associated with a personal history of attempted suicide in BD [63] and are ameliorated by lifetime lithium treatment [64]. Although the relationship between neural and behavioral alterations in BD needs further investigation [65], the altered control of prefrontal cortex over limbic structures may trigger the emotional hyper-reactivity in BD patients [66].

The deficit in right anterior cingulate cortex resulting from the VBM analysis was not confirmed at the regional level by the ROI analysis on Dataset1, probably due to the extension of the anterior cingulate region in the AAL atlas, which did not differentiate among its subregions. It should be mentioned that diverse findings came from previous region-based studies, which showed no GMV deficits in bilateral subgenual prefrontal cortex [67] and in right anterior cingulate cortex [68] in BD compared with HC. However, these results emerged from smaller patient samples compared to the present work.

It is worth noticing that our VBM analyses showed a right lateralization of the GMV deficits in BD patients, which is in line with the findings of previous structural [11] and functional [69] studies. This result is supported by the knowledge that right prefrontal cortex plays a key role in cognitive inhibition [70] and is corroborated by evidence that transcranial magnetic stimulation of this region represents an effective add-on treatment for BD [71, 72]. Such an asymmetry may also reflect the lower WM integrity in prefrontal-limbic, limbic and callosal tracts observed in BD compared with HC [30, 73–75]. However, Bellani and colleagues [76] investigated laterality effects in SCZ and BD compared with HC using a visuo-motor task, without finding any difference between BD and HC. Therefore, the issue of laterality in BD should be further investigated.

### 4.4. Differences between BD and SCZ

One of the main novelties of the present study concerns the direct comparison between SCZ and BD. Significant inter-diagnostic differences emerged only from the analysis of Dataset1, suggesting slighter differences between SCZ and BD patients than between each group of patients and HC. However, the VBM and ROI analyses of Dataset1 highlighted GMV deficits in SCZ compared to BD in the right insula and its operculum and in the thalami, especially in the left hemisphere.

So far, the limited number of studies that compared SCZ and BD, either directly or indirectly, usually found GMV deficits in SCZ compared to BD. Although the cortical regions interested by these deficits were rather heterogeneous across studies, our results partially confirm the previous ones.

In this respect, the volume losses in the insula and/or thalamus that we found in SCZ compared to BD have already been reported in the literature [3, 13–15, 51]. In the work from Bora and colleagues [51], such a difference was found to be related to the male dominance in the SCZ group, which also characterized our and other datasets. However, the hypothesis of a specific involvement of the thalamus in the pathophysiology of SCZ has been recently emphasized in the literature [77], corroborated by postmortem evidence of reduction in volume and number of neurons of the medial dorsal thalamic nucleus, the principal source of thalamic projections to the prefrontal cortex [78]. Pharmacological effects cannot be ruled out, because antipsychotics seem to exert effects on thalamic volumes [79]. The structural differences between SCZ and BD in the thalamus may also be reflected in the lower thalamic function of SCZ compared to BD that was reported in [80].

The further hypothesis of a specific role of the insula in SCZ pathology is supported by the exaggerated thalamic-insular functional connectivity characterizing SCZ compared to HC [81]. The above deficits may also relate to the lower metabolism in frontal, parietal and temporal WM tracts that emerged in SCZ compared to BD [82].

Differently from our study, GMV reductions in SCZ compared to BD were also described in the right lingual gyrus [12], amygdala, putamen and hippocampus [2] as well as in the cerebellum [83].

### 4.5. Comparison with other multicentric studies

To our knowledge, this is the one of the first multicentric studies on SCZ and BD with such a large number of patients and combining ROI and VBM analyses.

A very recent multicentric study that deserves mention was conducted by Ivleva and colleagues [12], who compared GMV in a large sample of patients with SCZ, PBD and schizoaffective disorder (SAD), their first-degree relatives and HC. In their study, GMV was investigated at the voxel-level and in 5-mm radius spheres centered on the VBM peak voxels. In line with our results, they showed broader GMV deficits in SCZ than in BD when compared to HC, as well as GMV deficits in BD in the cingulate and superior temporal cortices, mainly in the right hemisphere. It is worth noticing that Ivleva et al. contrasted GMV across DSM diagnoses and psychosis biotypes, providing evidence for the meaningful classification of patients based on neurobiological constructs. This represents an interesting perspective that should be investigated in the next future, when a larger sample of PBD patients will be available.

Another study that deserves mention [84] involved 13 Centers and around 500 subjects, either SCZ patients or HC, and produced VBM and ROI results on SCZ that were overall in agreement with ours. However, compared to our study, it was limited by the absence of BD patients, besides being based on a smaller sample of subjects distributed among a larger number of centers, which made the results more susceptible to confounding center effects.

### 4.6. Methodological issues

Our results were obtained by applying both voxel-based and region-based analyses, whose integration represents one of the novelties of our study. Here, the ROI analysis was introduced to inspect the reproducibility of the VBM findings at the regional level. It should be remarked that the ROI analysis was VBM-driven, as the selection of the AAL regions was based on VBM results, and that VBM and ROI results were not fully independent, as the ROI volume was computed on the same tissue density maps of the VBM study. However, the two approaches provide different information and have complementary advantages and disadvantages. In region-based approaches, the results reliability depends on the choice of the regions, which can be either manual or automatic, but the results are less sensitive to local imperfections than in voxel-based approaches. Therefore, in our study, the combination of VBM and ROI analyses allowed to investigate both local and global GMV differences and to provide more robust evidence of the neuroanatomical bases of BD and SCZ.

The choice to include the analysis of Dataset2, a subset of Dataset1, was motivated by the inhomogeneous characteristics of Dataset1. Although the GLM design modeled and removed the contribution of age, gender and center, the comparison between the results of Dataset1 and Dataset2 allowed to identify the most relevant results. The findings of Dataset2 were less significant compared to the ones of Dataset1 due to the smaller sample, but less vulnerable to confounding factors. A more robust validation may come from the analysis of an independent sample, which is encouraged in the next future.

The VBM and ROI results on both datasets showed an overall agreement, which demonstrates not only the reliability of the findings on the two datasets, but also the validity of the quantitative techniques that were applied. In both datasets, the site represented a relevant contributor: the different imaging parameters, types of MR scanner and head coils led to images with different spatial resolution, contrast and intensity, whose contribution was considered in the analysis.

In the main study design, we did not account for clinical information such as age of illness, psychopathological scores, pharmacological treatment and substance abuse, which may have an influence on the brain structural characteristics. This choice was motivated by the heterogeneity of available information and by the variety of treatments across the four centers. However, we investigated the contribution of a set of clinical variables on GMV in subsamples of patients, according to the available information. The results concerning the BD sample suggest that mood state, BD type and psychotic symptoms should have not influenced the results. However, we cannot exclude possible confounding effects due to 1) age of onset and medication in BD patients, 2) symptoms severity in SCZ patients. These limitations should be taken into account when interpreting the current results.

### 4.7. Future perspectives

The results of this large neuroimaging study demonstrate the importance of comparing BD and SCZ (between themselves and with HC) to gain new knowledge of the shared and specific neuroanatomical bases of the two disorders, bringing added value to the clinical management of BD and SCZ.

However, our results need to be confirmed by future investigations on large independent samples and should be integrated with the study of gender and age effects in both BD and SCZ. On the one hand, the investigation of brain sexual dimorphism in BD and SCZ can shed new light on the relationship between sexual differentiation processes and vulnerability to develop psychiatric disorders. On the other hand, the analysis of age-related neuroanatomical changes has potential to highlight any deviations from normal brain maturation shared by BD and SCZ or diagnosis-specific. These complex topics will be the object of a future dedicated investigation from the ENPACT group.

In the near future, we are interested in repeating the SCZ-BD comparison by using a larger BD sample, with a sufficient and balanced number of psychotic and non-psychotic patients, with the objective to identify any neurobiological characteristics associated with psychotic features across traditionally distinct categorical diagnoses.

## 5. Conclusions

Although SCZ and BD are conceptualized in a continuum of clinical phenotypes, the underlying neural mechanisms are still largely unknown. In the present study, structural MRI images of a large sample of subjects were analyzed with voxel-based and region-based techniques to identify GMV differences and similarities between SCZ and BD. The two disorders exhibited shared fronto-temporo-occipital GMV deficits in the right hemisphere, which support the hypothesis of a continuous GM endophenotype for SCZ and BD. Our study demonstrates that the two disorders are not completely dichotomous in terms of GM anatomy, but also suggest that SCZ is associated with brain structures that are more severely compromised in comparison with BD. A specific involvement of thalamus and insular cortex in SCZ is suggested, which needs further investigation. The results of our study provide a key piece of information for the comprehensive understanding of the two disorders, opening the door to advanced diagnostic and treatment strategies.

## Supporting information

S1 Results Results of the VBM analysis of Dataset2. (DOCX)

S1 Database Database information. Demographic and clinical information on Dataset1 and Dataset2.
(XLSX)

S1 Fig **Left: Results of the VBM analysis of Dataset2. Significant regions emerging from the SCZ<HC t-contrast (p<0.05, cFWE corrected). Right: Results of the ROI analysis of Dataset2. AAL regions with significant GMV differences between SCZ and HC (p<0.05, Bonferroni corrected).** VBM: voxel based morphometry. ROI: region of interest. AAL: Automated Anatomical Labeling. GMV: gray matter volume. SCZ: schizophrenia. HC: healthy controls. cFWE: cluster family wise error.
(TIFF)

S1 Table Statistics of the significant ROIs (p<0.05, cFWE corrected) of the VBM analysis of Dataset2. Degrees of freedom: [1 284]. t-stat threshold: 3.12. SCZ: schizophrenia. HC: healthy controls. FWE: family wise error. AAL: Automated Anatomical Labeling. R: right hemisphere. L: left hemisphere.
(DOCX)

S2 Table Significant AAL regions of the region-based analysis of Dataset2 (p<0.05, Bonferroni corrected). SCZ: schizophrenia. HC: healthy controls. AAL: Automated Anatomical Labeling.
(DOCX)
