# Supplementary material for: Common and distinct structural features of schizophrenia and bipolar disorder: The European Network on Psychosis, Affective disorders and Cognitive Trajectory (ENPACT) study
Source: PLoS One. 2017 Nov 14;12(11):e0188000. doi: 10.1371/journal.pone.0188000 (PMC5685634; doi:10.1371/journal.pone.0188000)
Supplement: S1 Results — (DOCX) [file pone.0188000.s001.docx]

# Supplementary results

## Voxel Based Morphometry (VBM) analysis

The VBM analysis performed on Dataset2 led to results that were in overall agreement with Dataset1 ones. However, in Dataset2, the only differences that survived the cluster family wise error (cFWE) correction were relative to schizophrenia (SCZ) patients compared to healthy controls (HC) (S1 Fig, left panel). The spatial parameters and statistics of the regions emerging from the SCZ<HC t-contrast (p<0.05, cFWE corrected) are listed in S1 Table. This analysis confirmed that SCZ patients are characterized by lower gray matter volume (GMV) than HC in bilateral orbitofrontal cortex, cingulate cortex, superior temporal cortex, insula, thalamus, hippocampus and precuneus. These regions were mainly included in the ones already emerged from Dataset1, except from the cluster in the bilateral precuneus. The GMV deficits in right superior temporal cortex and temporal pole of bipolar disorder (BD) patients compared to HC were confirmed at the uncorrected level, as well as the lower GMV in SCZ patients than in BD ones at the level of the insula.

## Region Of Interest (ROI) analysis

The ROI analyses of Dataset2 were performed on the anatomical regions from the VBM comparison between SCZ and HC (t-contrast: SCZ<HC, p<0.05, cFWE corrected), whose results survived the multiple comparison correction. The local GMV deficits were confirmed at the regional level with high significance (p<0.05, Bonferroni corrected) in the majority of the AAL regions (31 out of 54), mainly located in the frontal and temporal cortices of both hemispheres (S2 Table and S1 Fig, right panel).

**S1 Fig. Left: Results of the VBM analysis of Dataset2. Significant regions emerging from the SCZ<HC t-contrast (p<0.05, cFWE corrected). Right: Results of the ROI analysis of Dataset2. AAL regions with significant GMV differences between SCZ and HC (p<0.05, Bonferroni corrected)*.*** VBM: voxel based morphometry. ROI: region of interest. AAL: Automated Anatomical Labeling. GMV: gray matter volume. SCZ: schizophrenia. HC: healthy controls. cFWE: cluster family wise error.
