# Supplementary figures and images for: Common and distinct structural features of schizophrenia and bipolar disorder: The European Network on Psychosis, Affective disorders and Cognitive Trajectory (ENPACT) study
Source: PLoS One. 2017 Nov 14;12(11):e0188000. doi: 10.1371/journal.pone.0188000 (PMC5685634; doi:10.1371/journal.pone.0188000)

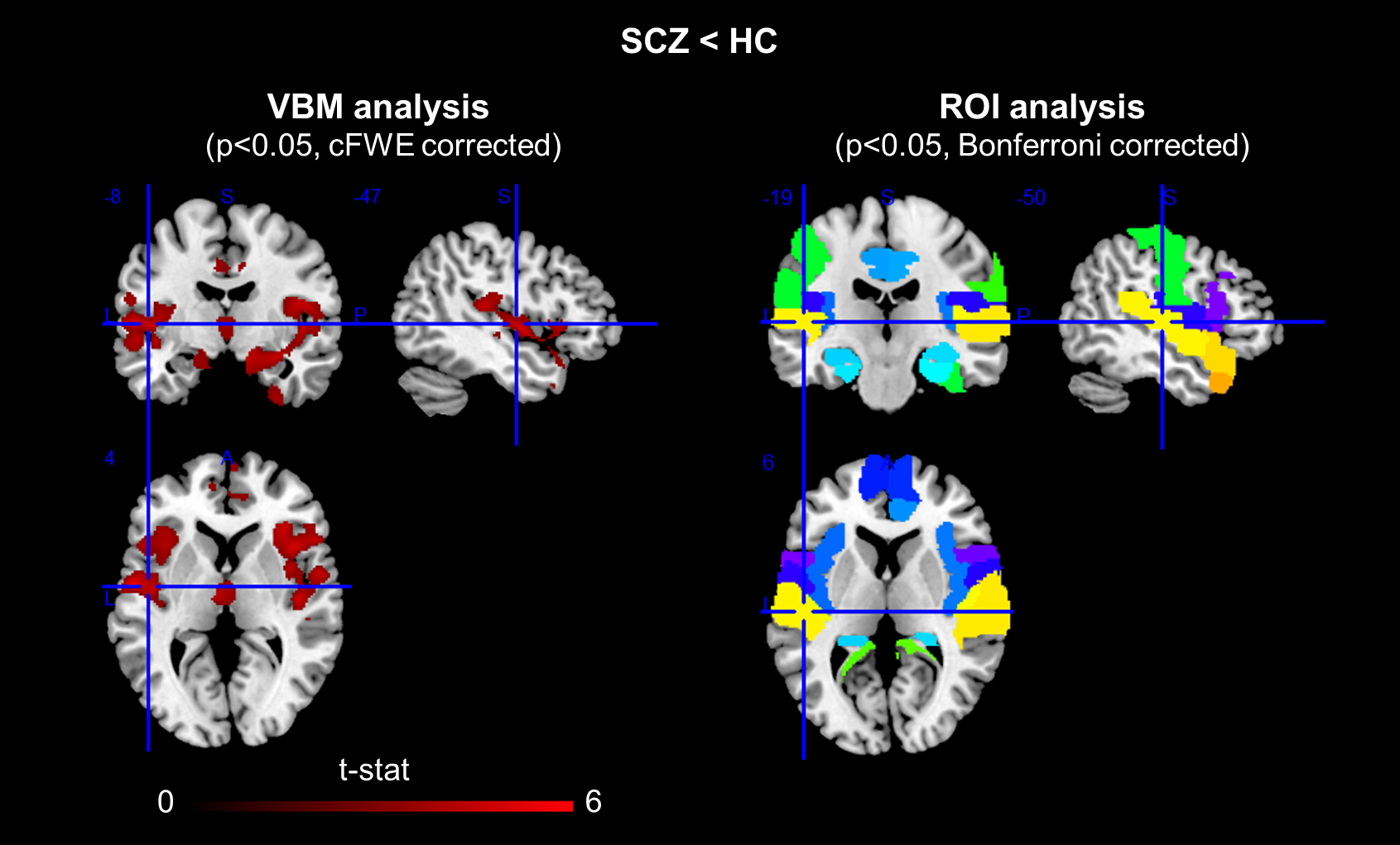

Supplement: S1 Fig — Left: Results of the VBM analysis of Dataset2. Significant regions emerging from the SCZ<HC t-contrast (p<0.05, cFWE corrected). Right: Results of the ROI analysis of Dataset2. AAL regions with significant GMV differences between SCZ and HC (p<0.05, Bonferroni corrected). VBM: voxel based morphometry. ROI: region of interest. AAL: Automated Anatomical Labeling. GMV: gray matter volume. SCZ: schizophrenia. HC: healthy controls. cFWE: cluster family wise error. (TIFF) [file pone.0188000.s003.tiff]
