# Supplementary material for: Common and distinct structural features of schizophrenia and bipolar disorder: The European Network on Psychosis, Affective disorders and Cognitive Trajectory (ENPACT) study
Source: PLoS One. 2017 Nov 14;12(11):e0188000. doi: 10.1371/journal.pone.0188000 (PMC5685634; doi:10.1371/journal.pone.0188000)
Supplement: S1 Table — Degrees of freedom: [1 284]. t-stat threshold: 3.12. SCZ: schizophrenia. HC: healthy controls. FWE: family wise error. AAL: Automated Anatomical Labeling. R: right hemisphere. L: left hemisphere. (DOCX) [file pone.0188000.s004.docx]

**S1 Table Statistics of the significant ROIs (p<0.05, cFWE corrected) of the VBM analysis of Dataset2. Degrees of freedom: [1 284]. t-stat threshold: 3.12.**

| **T contrast** | **cluster**  **p(FWE-corr)** | **# voxels** | **Peak T** | **x,y,z {mm}** | **AAL region** |
| --- | --- | --- | --- | --- | --- |
| **SCZ < HC** | <0.001 | 9595 | 5.44 | 54, 0, 2 | Superior temporal cortex, R |
|  | <0.001 | 5037 | 5.35 | -57, -5, 5 | Superior temporal cortex, L |
|  | <0.001 | 4055 | 4.91 | -2, 35, 47 | Superior frontal cortex, medial portion, L |
|  | 0.001 | 1218 | 4.74 | 12, 59, -3 | Orbitofrontal cortex, medial portion, R |
|  | 0.001 | 1221 | 4.56 | 3, -32, 42 | Mid cingulate cortex, R |
|  | 0.029 | 558 | 4.33 | 6, -12, 5 | Thalamus, R |
|  | 0.001 | 1070 | 4.21 | 9, -60, 38 | Precuneus, R |
|  | 0.037 | 524 | 3.9 | -14, -3, -18 | Parahippocampal gyrus, L |

SCZ: schizophrenia. HC: healthy controls. FWE: family wise error. AAL: Automated Anatomical Labeling. R: right hemisphere. L: left hemisphere.
