# Supplementary material for: Common and distinct structural features of schizophrenia and bipolar disorder: The European Network on Psychosis, Affective disorders and Cognitive Trajectory (ENPACT) study
Source: PLoS One. 2017 Nov 14;12(11):e0188000. doi: 10.1371/journal.pone.0188000 (PMC5685634; doi:10.1371/journal.pone.0188000)
Supplement: S2 Table — SCZ: schizophrenia. HC: healthy controls. AAL: Automated Anatomical Labeling. (DOCX) [file pone.0188000.s005.docx]

**S2** **Table Significant AAL regions of the region-based analysis of Dataset2 (p<0.05, Bonferroni corrected).**

| **T contrast** | **AAL clusters**  **Left hemisphere** | **AAL clusters**  **Right hemisphere** |
| --- | --- | --- |
| **SCZ < HC** | inferior opercular frontal cortex , rolandic operculum, superior medial frontal cortex, insula, mid cingulate cortex, hippocampus, parahippocampal gyrus, amygdala, postcentral gyrus, precuneus, Heschl’s gyrus, superior temporal cortex , temporal pole. | Inferior opercular frontal cortex , rolandic operculum, superior medial frontal cortex, superior orbitofrontal cortex,  insula, anterior and mid cingulate cortex, hippocampus, parahippocampal gyrus, amygdala, fusiform gyrus, supramarginal gyrus, precuneus, Heschl’s gyrus, superior temporal cortex, temporal pole. |

SCZ: schizophrenia. HC: healthy controls. AAL: Automated Anatomical Labeling.
